# CRISPR-Based Engineering of Mesenchymal Stem Cells: Functional Enhancement, Translational Challenges, and Clinical Perspectives

**DOI:** 10.3390/cells15212029

**Published:** 2026-11-06

**Authors:** Geofanny Facicilia, Cynthia Retna Sartika, Yuni Elsa Hadisaputri, Tina Rostinawati

**Affiliations:** 1 Department of Pharmaceutical Biology, Faculty of Pharmacy, Universitas Padjadjaran, Jatinangor 45363, Indonesia; geofanny02@gmail.com (G.F.); t.rostinawati@unpad.ac.id (T.R.); 2 Prodia StemCell Indonesia, DKI Jakarta, Jakarta 13410, Indonesia; c.sartika@gmail.com

**Keywords:** mesenchymal stem cells, CRISPR/Cas9, CRISPR activation, gene engineering, cell therapy, functional potency

## Abstract

Mesenchymal stem cells (MSCs) are promising platforms for regenerative and immunomodulatory therapies, but their clinical performance is limited by donor heterogeneity, variable potency, and limited persistence. CRISPR-based engineering provides programmable approaches to modify MSC function through genome editing and transcriptional regulation. This review examines CRISPR/Cas9-mediated knockout and knock-in, dCas9-based transcriptional regulation, and emerging base-, prime-, and epigenome-editing technologies for MSC engineering. These approaches can enhance survival and stress resistance, homing and tissue retention, paracrine and extracellular vesicle activity, immunomodulation, immune compatibility, and lineage-associated functions. Key translational challenges include efficient delivery, engineered-population heterogeneity, genomic safety, immunogenicity, phenotypic stability, scalable manufacturing, and mechanism-linked potency assessment. The appropriate engineering strategy should therefore be selected according to the intended therapeutic mechanism and product development requirements. Clinical translation will ultimately depend on demonstrating reproducible functional enhancement, manufacturing consistency, genomic and phenotypic stability, and validated therapeutic potency.

## 1. Introduction

Mesenchymal stem cells (MSCs) are considered promising therapeutic platforms due to their immunomodulatory, paracrine, and tissue-reparative properties [1]. However, clinical performance may be affected by donor and tissue-source heterogeneity, passage-dependent phenotypic changes, variable functional potency, and limited persistence after administration. Traditional preconditioning approaches may alter certain components of the MSC secretome, whereas CRISPR/Cas-based engineering offers a programmable method for modification of specific genes and regulatory pathways [2]. It is important to point out that this ability should not be interpreted as being intrinsically better than other engineering approaches; its merit is defined by the reproducibility of a functional gain with therapeutic relevance resulting from a particular molecular intervention.

CRISPR/Cas9 can be used for permanent gene disruption or targeted sequence modification, but double-strand-break (DSB)-based editing raises additional concerns about off-target cleavage, DNA-damage responses, and genomic stability during ex vivo expansion [3]. At the same time, delivery is still challenging in primary MSCs, and immune recognition of bacterial Cas proteins may be relevant in case of continued editor expression [4,5]. Thus, CRISPR activation (CRISPRa) utilizes nuclease-inactivated Cas9 (dCas9) to upregulate transcription from endogenous loci without inducing DSBs and is complementary when therapeutic benefit can be derived from increased gene expression without permanent sequence change [6]. This review provides a critical appraisal of CRISPR-based strategies for engineering MSCs and links genome editing and transcriptional regulation to functional enhancement, challenges in delivery and manufacturing, safety, potency assessment, regulatory translation, and disease-specific applications.

Clinical justification for CRISPR engineering should be made using an appropriate unmodified-MSC comparator, rather than assumed based on editing efficiency alone. Such specific measures may enhance survival, trafficking, secretory activity or immune compatibility, depending on the indication, but direct functional and safety comparisons are necessary to confirm these benefits. The intrinsic donor- and tissue-dependent heterogeneity can be increased by variable editing outcomes and post-editing selection, which may complicate identity, phenotypic consistency, potency and genomic safety [1,2,7,8]. Thus, mechanism-linked potency assays, population-level characterization and evaluation of longitudinal stability should be used to demonstrate clinically meaningful advantages [9].

## 2. CRISPR-Engineered Strategies for MSCs

CRISPR-based approaches provide distinct strategies for modifying MSC functions, with different implications for editing outcomes, precision, and safety. This section discusses CRISPR/Cas9-mediated genome editing, the DNA repair pathways that determine editing outcomes, and dCas9-based transcriptional regulation [10,11].

### 2.1. CRISPR/Cas9-Mediated Genome Editing

The classical CRISPR/Cas9 genome-editing system primarily comprises a Cas9 endonuclease and a single-guide RNA (sgRNA) that guides the nuclease to a complementary genomic sequence. For target recognition, a protospacer-adjacent motif (PAM) is required, normally 5’-NGG-3’ for *Streptococcus pyogenes* Cas9 (SpCas9), adjacent to the target sequence [12]. Upon recognition by the PAM, the local DNA is unwound to enable the sgRNA spacer sequence to hybridize to the complementary DNA strand, leading to the formation of an RNA–DNA heteroduplex and an associated R-loop structure that stabilizes the Cas9–DNA complex [13]. Following target recognition, the two catalytic domains of Cas9 undergo conformational activation. The HNH nuclease domain cleaves the DNA strand complementary to the sgRNA, whereas the RuvC domain cleaves the opposite strand, generating a double-strand DNA break (DSB) approximately three nucleotides upstream of the PAM (Figure 1A) [12,13]. The generation of this site-specific DSB is the central event that enables conventional CRISPR/Cas9-mediated genome editing because the resulting DNA lesion is processed by endogenous cellular repair pathways, thereby converting sequence-specific cleavage into a permanent genomic alteration. The biological outcome of Cas9 cleavage therefore depends not only on target recognition but also on how the cell repairs the resulting DSB. Repair through non-homologous end joining (NHEJ) commonly produces small insertions or deletions that can disrupt coding sequences and generate gene knockout, whereas homology-directed repair (HDR) can be exploited to introduce defined sequence changes when an appropriate donor template is provided [14,15,16,17,18]. Thus, the same Cas9 platform can support both loss-of-function editing and precise sequence modification depending on the repair pathway and experimental design.

A major advantage of CRISPR/Cas9 over earlier programmable nuclease systems such as zinc-finger nucleases (ZFNs) and transcription activator-like effector nucleases (TALENs) is the separation of target recognition from nuclease-protein redesign. Whereas ZFNs and TALENs generally require modification of DNA-binding proteins for each new locus, Cas9 can be redirected by altering the approximately 20-nucleotide targeting sequence within the sgRNA [19,20,21]. This modularity also facilitates multiplexed editing, in which several genomic loci can be targeted within the same engineering workflow. This flexibility is exploited for MSC engineering, which can permanently disrupt or modify genes involved in apoptosis, migration, differentiation, immune recognition, and therapeutic secretion. However, DSB formation also presents potential safety concerns. Off-target effects are possible because Cas9 can tolerate some mismatches between the sgRNA and the DNA target [13,22]. In addition, DSBs can activate cellular DNA-damage responses, and prolonged expansion after editing may provide opportunities for rare genomic alterations to become enriched within the engineered population [3]. These issues are particularly relevant to MSC-based products because editing is followed by ex vivo expansion before therapeutic administration.

Several approaches have therefore been developed to increase editing precision. High-fidelity variants of Cas9 such as eSpCas9, SpCas9-HF1 and HiFi Cas9 reduce non-specific DNA interactions and thus increase discrimination between intended and unintended targets [22]. Cas9 nickases offer another strategy by making single-strand breaks that can be paired to increase target specificity. Transient delivery of pre-assembled Cas9 ribonucleoprotein complexes provides an alternative approach to limit the duration of intracellular nuclease exposure [5]. However, as conventional Cas9 editing ultimately relies on DNA cleavage, understanding the cellular pathways that repair the resulting DSB is critical to predicting and controlling editing outcomes in MSCs.

### 2.2. NHEJ and HDR as Determinants of CRISPR Editing Outcomes in MSCs

Following a Cas9-induced DSB, the final editing outcome is largely determined by the cellular DNA-repair machinery. Two major repair pathways are commonly exploited in CRISPR/Cas9 genome engineering: non-homologous end joining (NHEJ) and homology-directed repair (HDR). Although both pathways respond to DNA breaks, they differ substantially in mechanism, cell-cycle dependence, and editing outcome. Consequently, the choice between NHEJ- and HDR-based editing is closely linked to whether the intended MSC modification requires gene disruption or precise sequence alteration [14,15,16,17,18].

NHEJ repairs DSBs without requiring an external homologous DNA template and is active throughout much of the mammalian cell cycle. The pathway is initiated by recognition of exposed DNA ends by the Ku70/Ku80 complex, followed by recruitment of DNA-dependent protein kinase and additional end-processing and ligation factors [14]. Because broken DNA ends can undergo limited processing before re-ligation, repair frequently produces small insertions or deletions at the cleavage site. When these indels occur within a protein-coding region, they can introduce frameshift mutations or disrupt functionally important sequences, making NHEJ particularly useful for gene-knockout applications. The broad cell-cycle availability of NHEJ is particularly relevant to MSCs. Primary MSC cultures are heterogeneous in proliferative state, and proliferative capacity can change according to tissue source, donor characteristics, culture conditions, and passage number [15]. Because NHEJ does not depend on S/G2-phase homologous templates, it can remain accessible in cells with relatively limited proliferative activity. Consequently, many CRISPR/Cas9 MSC-engineering strategies employ NHEJ when the therapeutic objective is permanent disruption of a target gene, such as an apoptosis-associated, differentiation-associated, or immunological regulator.

HDR follows a fundamentally different mechanism. After DNA-end processing, homologous sequence information is used to reconstruct the damaged locus, allowing for accurate sequence replacement or insertion. During canonical homologous recombination, resected single-stranded DNA is processed through repair intermediates involving homologous-sequence recognition and RAD51-associated strand invasion [14,16,17,18]. Experimental CRISPR workflows can exploit this mechanism by supplying an exogenous donor template containing the desired sequence flanked by regions homologous to the target locus. HDR can therefore support precise knock-in, sequence correction, or targeted integration of therapeutic genetic elements. Unlike NHEJ, HDR is strongly associated with the S and G2 phases of the cell cycle, when homologous DNA templates and recombination machinery are most available [16,17,18]. This restriction presents an important challenge for MSC engineering because primary MSCs may proliferate relatively slowly, and prolonged ex vivo expansion can further alter proliferative activity. HDR efficiency therefore depends on more than nuclease activity alone and can be affected by cell-cycle status, tissue source, donor-template configuration, delivery method, and the physiological state of the MSC population.

Precise targeted integration, however, has been demonstrated in primary MSCs. Srifa et al. delivered Cas9 ribonucleoprotein together with adeno-associated virus serotype 6 (AAV6) donor templates and obtained targeted integration efficiencies of 12.4 ± 3.0% at the CCR5 locus and 20.8 ± 5.0% at RANKL in human bone marrow-derived MSCs [7]. Equivalent approaches were examined on the HBB locus with other MSC sources, with integration efficiencies of 40.8 ± 6.2% and 32.1 ± 12.8% for adipose- and umbilical cord blood-derived MSCs [7]. These results demonstrate the feasibility of HDR-mediated knock-in in primary MSCs and highlight the impact of tissue source and delivery conditions on editing efficiency.

NHEJ and HDR should therefore be regarded as complementary rather than competing mechanisms for MSC engineering. NHEJ is generally more practical when the therapeutic objective requires permanent gene disruption, whereas HDR is necessary when precise insertion, replacement, or sequence correction is required. Importantly, however, neither pathway is intrinsically preferable for all MSC applications. The editing strategy should be selected according to the intended mechanism of action, required duration of the modification, acceptable genomic risk, and downstream manufacturing requirements. When the therapeutic objective can instead be achieved by increasing or decreasing endogenous gene expression without changing the underlying DNA sequence, dCas9-based transcriptional regulation provides an alternative to DSB-dependent editing.

### 2.3. dCas9-Based Transcriptional Regulation and CRISPR Activation

Catalytically inactive Cas9 (dCas9) extends the CRISPR platform beyond permanent genome editing to programmable regulation of endogenous gene expression. dCas9 is produced by inactivating the RuvC and HNH nuclease domains of Cas9, usually by introducing mutations D10A and H840A, respectively. These mutations eliminate DNA-cleavage activity but maintain an ability to recognize specific genomic loci via sgRNA, converting Cas9 from a nuclease to a programmable DNA-binding scaffold [6]. When dCas9 is fused to transcriptional activators, the system is called CRISPR activation (CRISPRa). Unlike nuclease-active CRISPR/Cas9-mediated genome editing, CRISPRa does not intentionally change the nucleotide sequence of the target gene. Rather, it recruits transcriptional machinery to the promoter or regulatory regions to increase transcription from the endogenous locus, without inducing Cas9-mediated DNA double-strand breaks. Early systems utilized dCas9 fused to the VP64 activation domain, but later platforms were engineered to enhance transcriptional activity (Figure 1B). Examples include dCas9-VPR which consists of VP64, p65 and Rta activation domains and the Synergistic Activation Mediator (SAM) system, which utilizes dCas9-VP64 and modified sgRNAs that recruit additional transcriptional activators [23]. Also, dCas9 can be fused to epigenetic effectors such as p300 to change local chromatin accessibility and increase transcriptional activation [24]. A major conceptual advantage of CRISPRa is that gene expression is activated from the endogenous genomic locus, and not from a separately introduced cDNA cassette. Such distinction can preserve some native promoter context, transcript processing and regulatory feedback that can be lost with constitutive transgene overexpression [24]. CRISPRa does not cause a Cas9-induced DSB and therefore bypasses DNA-break-dependent repair and the chance of indel formation at the intended target. However, this does not eliminate the need for specificity assessment as off-target binding or activation by dCas9 at non-target regulatory regions is still possible.

Transcriptional regulation is of relevance for MSC engineering when therapeutic improvements can be achieved by increasing the activity of an endogenous pathway instead of permanently modifying the DNA sequence. CRISPRa-mediated activation of PDGFR-β in adipose-derived MSCs enhanced survival, migration, and proangiogenic paracrine activity in diabetic-wound models [25]. Activation of TSG-6 has also been linked to the modified anti-inflammatory activity and cargo of MSC-derived extracellular vesicles [26]. In primary human MSCs, a lentiviral-delivered SAM system was able to activate transcriptionally inactive CEBPA and CIITA loci and trigger downstream HLA-DR surface expression [27]. These studies show that endogenous transcriptional regulation can modulate therapeutically relevant MSC functions without direct perturbation of the target gene. Thus, the limiting factor for CRISPRa is not DNA cleavage, but delivery and expression of the regulatory machinery. Conventional Cas9 is significantly smaller than dCas9-effector constructs, particularly in multi-component systems like VPR and SAM, imposing further constraints on vector design and delivery to primary MSCs [6,28]. The reliable expression has often been based on the integration of vectors such as lentivirus, which can support continuous activity, but also introduces additional concerns regarding genomic integration, long-term expression and silencing of the transgene [27]. In an effort to minimize the duration and the genomic footprint of CRISPR components, non-integrating strategies such as mRNA, peptides and other transient delivery approaches are being explored [28,29]. Importantly, dCas9 can also be fused to transcriptional repressors (CRISPR inhibition, CRISPRi) or chromatin-modifying enzymes for locus-specific epigenetic regulation. Such approaches extend the CRISPR-based MSC engineering beyond the simple gene knockout or gene activation. However, their wider translational potential is discussed further in Section 8, as their application in primary therapeutic MSCs is not as developed as conventional Cas9 editing and CRISPRa.

### 2.4. Selecting the Appropriate CRISPR Engineering Strategy for MSCs

The type of CRISPR engineering modality should be dictated by the biological change required to achieve the desired therapeutic mechanism of action. The standard Cas9-mediated editing is most useful when a permanent change in the sequence is required, such as when an inhibitory or immunogenic gene is disrupted, a pathogenic sequence is corrected, or a therapeutic element is inserted in a targeted gene sequence. However, dCas9-based transcriptional regulation may be more appropriate in cases where the desired phenotype can be obtained by upregulating or downregulating endogenous gene expression without permanent modification of the underlying DNA sequence. Thus, these approaches are complementary rather than competing strategies. Nuclease-mediated editing can produce stable knockout or knock-in phenotypes, but it requires handling of DSB-associated DNA-damage responses, repair heterogeneity and off-target cleavage. CRISPRa and CRISPRi prevent Cas9-induced DSBs but have other limitations, including the increased size of dCas9-effector complexes, differential accessibility of the chromatin, dependence on continuous expression of the regulators, and the possibility of transcriptional heterogeneity in a population of MSCs [6,23,28].

The optimal strategy also depends on the length and magnitude of the desired therapeutic effect. Permanent editing would be useful if a permanent change in an intrinsic MSC property is required in manufacturing and after administration. Conversely, when therapeutic benefit relies on a controlled enhancement in survival, migration, immunomodulation or paracrine activity, transcriptional regulation might be more suitable. In both cases, the engineering outcome should ultimately be evaluated at the functional level, not by editing efficiency or changes in gene expression alone. Accordingly, CRISPR-based MSC engineering should be viewed as a spectrum of programmable interventions ranging from permanent genome modification to reversible or tunable gene regulation. Selection of the appropriate modality should integrate target biology, desired duration of effect, delivery feasibility, genomic safety, population consistency, and compatibility with the intended manufacturing process.

## 3. Functional Enhancement in MSCs

CRISPR-based engineering provides an opportunity to modify specific biological properties of mesenchymal stem cells (MSCs) that influence their therapeutic performance. Rather than altering MSC identity, genome editing and transcriptional regulation can be directed toward defined functional limitations, including poor survival after transplantation, inefficient tissue homing, variable paracrine activity, incomplete immunomodulatory responses, or undesirable differentiation outcomes [1,2]. The therapeutic relevance of an engineered MSC therefore depends on whether the selected molecular modification produces a measurable improvement in the function required for a particular indication. The major functional objectives reported for CRISPR-engineered MSCs can be grouped into survival and stress resistance, homing and tissue retention, paracrine and extracellular vesicle activity, immunomodulation and immune compatibility, and lineage- or matrix-associated functions. These categories are interconnected rather than independent; for example, improved survival may increase the duration of paracrine activity, whereas enhanced homing may increase the local concentration of immunomodulatory or regenerative signals. Representative targets and reported functional outcomes are summarized in Table 1, while the major functional outputs of CRISPR-engineered MSCs are illustrated in Figure 2.

### 3.1. Survival and Stress Resistance

Limited survival after administration is an important constraint in MSC-based therapy, particularly when cells are introduced into ischemic, inflammatory, or otherwise hostile tissue environments. Hypoxia, oxidative stress, nutrient deprivation, and inflammatory signaling can activate apoptotic pathways and reduce the number of viable cells available to exert therapeutic effects. CRISPR-based engineering has therefore been investigated as a means of increasing resistance to cellular stress by either disrupting pro-apoptotic pathways or enhancing endogenous pro-survival signaling.

Gene disruption represents one approach to improving MSC survival. CRISPR/Cas9-mediated disruption of KEAP1 in human bone marrow-derived MSCs enhanced NRF2-associated antioxidant defenses, cellular viability under oxidative stress, and vascular regeneration in a mouse ischemia model [32]. In this setting, targeted disruption of a negative regulator of antioxidant signaling can provide sustained protection from oxidative injury. Transcriptional activation offers an alternative when complete gene disruption is unnecessary. CRISPRa-mediated activation of PDGFR-β in adipose-derived MSCs also increased survival and paracrine activity, with improved diabetic wound healing in mice [25]. This approach illustrates an important distinction between genome disruption and transcriptional enhancement: both may improve survival, but they act through different molecular mechanisms and may differ in the durability, magnitude, and safety profile of the resulting phenotype. Importantly, improved cell survival should not automatically be interpreted as improved therapeutic potency. Excessive resistance to apoptosis or modification of pathways involved in growth control may also alter normal cell turnover and therefore requires careful evaluation of proliferation, senescence, genomic stability, and long-term phenotype. Survival-enhancing strategies should consequently be assessed together with the therapeutic function that the engineered MSC is intended to provide.

### 3.2. Homing, Migration, and Tissue Retention

The therapeutic activity of MSCs can also be limited by inefficient trafficking to the target tissue and poor retention after systemic or local administration. MSC migration is influenced by chemokine gradients, adhesion molecules, and interactions with vascular and extracellular matrix components. Engineering these pathways may therefore increase the proportion of administered cells that reach or remain within a therapeutically relevant site. The CXCR4-SDF-1α axis is one of the most widely studied pathways for MSC homing. In a rat myocardial-infarction model, retroviral CXCR4 overexpression improved MSC homing to ischemic myocardium and functional outcomes; this provides evidence for CXCR4 as a target but is not an example of CRISPR engineering [33]. Other strategies have focused on chemokine receptors or adhesion-related molecules that promote inflammatory recruitment, such as CCR7 and PSGL-1-related mechanisms [1,34].

Another documented CRISPR strategy is Cas9–AAV6-mediated knock-in of PSGL-1 and FUT7 in human adipose-derived MSCs, which improved platelet interactions, adhesion, and homing to injured skin in mice [34]. While chemokine-receptor engineering mainly affects directional migration, engineering of adhesion may affect interactions with vascular surfaces or the extracellular matrices once the MSCs have arrived at the target tissue. Therefore, these functions should be distinguished, although both contribute to the more general concept of MSC homing. Enhanced migration alone, however, does not ensure therapeutic benefit. An MSC that reaches the target tissue must retain viability and deliver the intended regenerative, paracrine, or immunomodulatory function. Homing-related engineering should therefore be evaluated using functional migration or adhesion assays together with downstream potency measurements rather than being assessed solely by receptor-expression levels.

### 3.3. Paracrine, Angiogenic, and Extracellular Vesicle Functions

Many therapeutic effects attributed to MSCs are mediated through paracrine signalling rather than permanent engraftment or direct tissue replacement. MSCs secrete cytokines, growth factors, chemokines, and extracellular vesicles (EVs) that can influence angiogenesis, inflammation, immune-cell activity, and tissue repair [1,2]. CRISPR-based engineering can therefore be used to alter the quantity or composition of these secreted products and potentially strengthen specific therapeutic mechanisms.

Angiogenic enhancement provides a representative example. E-selectin-modified MSCs generated using an AAV-based, non-CRISPR approach have shown improved angiogenesis and wound repair in an ischemic-wound model, providing a relevant functional comparator for future CRISPR-based strategies [35]. In this setting, engineering is directed not primarily toward increasing MSC survival itself, but toward increasing the biological activity delivered by the engineered cells to surrounding tissues. CRISPR-based regulation can also influence anti-inflammatory and extracellular-vesicle-associated functions. CRISPRa-mediated activation of TSG-6 has been associated with changes in the activity and cargo of MSC-derived EVs in IL-1β-stimulated human intervertebral-disc cells [26]. Importantly, although EV cargo was altered, superiority of engineered-cell EVs over control EVs was not established in that study. These findings are particularly relevant because they demonstrate that engineering the parental MSC can alter not only intracellular gene expression but also downstream cell-free therapeutic outputs.

This relationship between cellular engineering and secreted products introduces an additional layer of product characterization. A molecular change within an MSC does not necessarily predict the magnitude or composition of its secretome or EV output. When the intended mechanism of action depends on secreted mediators, functional evaluation should therefore extend beyond confirmation of the genetic modification to include relevant secreted factors, EV characteristics, and mechanism-linked biological activity.

### 3.4. Immunomodulation and Immune Compatibility

Immunological engineering of MSCs can serve two conceptually different purposes. The first is to enhance the ability of MSCs to regulate recipient immune responses as part of their therapeutic mechanism. The second is to reduce immune recognition of the MSC product itself, particularly in an allogeneic setting. Although these objectives can involve overlapping molecular pathways, they should be distinguished when defining the purpose and potency of an engineered product.

Therapeutic immunomodulation can be enhanced by modifying pathways associated with anti-inflammatory or immune-regulatory activity. Targets discussed in MSC-engineering studies include TSG-6, IDO-associated mechanisms, and related immunomodulatory pathways [2,26]. CRISPR-based activation or modulation of these pathways may strengthen the ability of MSCs to alter inflammatory signalling or immune-cell responses without necessarily changing their compatibility with the recipient immune system. Immune compatibility engineering instead focuses on reducing recognition of administered MSCs. Modification of antigen presentation pathways, including B2M- and CIITA-associated mechanisms, has been investigated to alter major histocompatibility complex (MHC)-dependent recognition [31,36]. In human umbilical cord-derived MSCs, targeted B2M disruption combined with a B2M–HLA-G fusion gene knock-in reduced susceptibility to both allogeneic T-cell and NK-cell responses in experimental assays [37]. However, loss of classical MHC class I can increase susceptibility to natural killer (NK)-cell cytotoxicity and may compromise immunosuppressive potency; this has been observed following MHC class I suppression in human bone marrow-derived MSCs [38]. Strategies aimed at reducing allogeneic immune recognition must therefore consider both adaptive and innate immune responses rather than focusing exclusively on T-cell recognition [31,39]. This distinction is important for therapeutic design. Increasing immunosuppressive activity and reducing immunogenicity are not equivalent outcomes, and modification of one pathway may influence the other. An engineered MSC intended to evade recipient immunity should consequently be assessed for preservation of its desired therapeutic functions, while an MSC engineered for stronger immunomodulation should still be evaluated for unintended changes in immune recognition.

### 3.5. Lineage and Matrix-Associated Functions

CRISPR-based engineering can also be used to modify MSC differentiation and extracellular-matrix-associated functions, particularly in musculoskeletal and regenerative medicine applications. In these settings, the therapeutic objective differs from survival or immunomodulation because the engineered phenotype is intended to influence lineage commitment, matrix composition, or the quality of regenerated tissue. Differentiation-associated transcriptional regulators provide several potential engineering targets. CRISPR/Cas9-mediated RUNX2 knockout has been used to modify MSC-derived cartilage matrices [30], while combined dCas9-based SOX9 activation and PPARγ repression in rat bone marrow-derived MSCs promoted chondrogenesis and calvarial bone regeneration [40]. Because these regulators participate in interconnected differentiation networks, their modification may influence more than one lineage programme and should therefore be evaluated beyond a single molecular marker. CRISPR/Cas9 engineering has also been used to modify matrix-associated characteristics of MSC-derived tissues. Editing of *RUNX2* and *VEGF* in MSC-derived engineered extracellular matrices has been explored as a strategy to alter hypertrophic differentiation, vascularization, and ectopic ossification in osteochondral repair models [30]. Such approaches illustrate how CRISPR engineering may be used not only to change the behaviour of the MSC itself but also to influence the structure and biological properties of the tissue environment generated by the engineered cells. Non-CRISPR studies of amnion-derived MSC hepatic differentiation demonstrate additional source-dependent lineage potential [41].

For lineage-directed applications, successful editing should therefore be evaluated using functional differentiation and matrix quality endpoints rather than gene expression changes alone. Excessive enhancement in one differentiation pathway may also produce undesirable outcomes, such as hypertrophy or inappropriate tissue formation. The desired phenotype must consequently be defined according to the intended regenerative application. Collectively, these functional categories illustrate that the value of CRISPR engineering in MSCs lies not in editing efficiency alone but in the ability to generate a defined and therapeutically relevant phenotype. Survival, homing, paracrine activity, immunomodulation, immune compatibility, and differentiation are also biologically interconnected, meaning that improvement in one function may alter another. Selection of an engineering target should therefore begin with the intended mechanism of action and be accompanied by functional assays capable of demonstrating that the molecular modification translates into the desired therapeutic output. This principle also creates an important link between functional engineering and the delivery, manufacturing, safety, and potency considerations discussed in the following sections.

## 4. Delivery and Manufacturing Challenges

Translation of CRISPR-engineered mesenchymal stem cells (MSCs) requires more than successful molecular modification. Delivery efficiency, persistence of the engineering system, cellular heterogeneity, and scalability all influence whether an engineered MSC population can be manufactured reproducibly while retaining its identity and intended therapeutic function (Figure 3). These considerations also differ according to the engineering modality. Nuclease-mediated editing generally benefits from transient editor exposure, whereas dCas9-based transcriptional regulation may require sustained or repeated expression of larger regulatory components. Accordingly, delivery and manufacturing strategies should be designed around the characteristics of the final MSC product rather than editing efficiency alone [23,28].

### 4.1. Delivery of CRISPR Components into MSCs

Efficient intracellular delivery remains one of the major practical barriers to CRISPR engineering in primary MSCs. Available cargos include plasmid DNA, messenger RNA (mRNA), pre-assembled Cas9 ribonucleoprotein (RNP) complexes, donor DNA templates, and larger dCas9-effector systems, each of which differs in size, intracellular persistence, trafficking requirements, and potential cellular stress [29,42]. Primary MSCs are generally less permissive to non-viral transfection than commonly used immortalized cell lines, and delivery conditions must therefore be optimized to achieve sufficient engineering efficiency without compromising cell viability, proliferation, or phenotype [28,29]. The physical form of the CRISPR cargo also influences the duration and potential biological consequences of editor exposure.

Plasmid-based delivery can support prolonged intracellular expression but introduces a DNA intermediate and may provide less predictable expression between cells (Figure 4). mRNA delivery avoids genomic integration and generally produces transient protein expression, whereas RNP delivery introduces the Cas9–sgRNA complex directly and can further shorten the duration of nuclease activity [5,29]. Transient approaches are particularly attractive for conventional Cas9 editing because limiting editor persistence may reduce opportunities for unintended cleavage while avoiding stable introduction of the editing machinery. Electroporation and nucleofection are among the most widely used non-viral methods for delivering CRISPR components to primary cells. These methods can deliver plasmid, mRNA or RNP cargo directly into the cell but can also cause membrane damage, stress responses or loss of viability if electrical parameters are not properly optimized [29,42,43,44]. Lipid- and peptide-based systems offer further non-viral alternatives, though their efficiency in primary MSCs is dependent on formulation and cargo. Furthermore, cell-penetrating peptides such as RALA have been investigated to deliver Cas9- or dCas9-related elements into primary MSCs [29]. Viral vectors have greater delivery efficiency in many primary cell settings but have different translational considerations. Because lentiviral vectors can transduce both dividing and non-dividing cells efficiently and carry relatively large expression cassettes, they are ideally suited for stable dCas9-based regulatory systems. However, there are insertion-related and long-term expression issues of genomic integration [45,46]. Adeno-associated virus (AAV) is mostly non-integrating and has been widely used as a donor-template delivery platform, but its ~4.7-kb packaging capacity limits its applicability for large multi-component CRISPRa systems [47]. Adenoviral vectors can carry more cargo and provide transient expression but can also evoke stronger innate immune responses [46].

For viral CRISPR delivery, reported insert-size limits are approximately 4.7 kb for conventional AAV, about 8–10 kb for lentiviral vectors, and approximately 8–10 kb for standard first-generation adenoviral vectors; specialized helper-dependent adenoviral systems can accommodate up to approximately 36 kb. These are approximate vector-dependent packaging ranges, not universal limits for all designs [48]. By contrast, electroporation of plasmid DNA, mRNA, or Cas9 RNP does not have an equivalent fixed viral packaging ceiling: practical constraints arise from DNA-plasmid length, mRNA size and integrity, protein–RNA-complex dimensions, formulation, delivery efficiency, and MSC viability. Larger plasmids or dCas9-effector constructs may be more difficult to deliver consistently, while RNP delivery is limited mainly by protein-complex delivery and cell tolerance rather than a nucleotide packaging cap [29,48].

CRISPRa introduces an additional delivery challenge because dCas9-VPR, SAM-associated components, selectable markers, and sgRNA expression elements can collectively produce relatively large constructs [6]. Lentiviral delivery has consequently been used in primary-MSC CRISPRa studies. For example, SAM-mediated activation of CEBPA and CIITA was achieved in primary MSCs using lentiviral delivery, whereas a corresponding transposon-based strategy was less effective in the same study [27]. This illustrates an important translational trade-off: persistent delivery can improve the proportion and duration of engineered cells but may increase the genomic footprint and persistence of foreign components. High editing or transduction efficiency should therefore not be considered an independent measure of platform suitability. Lentiviral studies have reported transduction efficiencies of approximately 74–89% depending on construct complexity and selection conditions [49], whereas Cas9 RNP combined with donor-template approaches produced locus- and MSC-source-dependent integration efficiencies of approximately 12–40% [7]. Chemical modification of sgRNAs may further enhance guide stability and editing performance [50]. However, these values are platform- and study-specific and should always be interpreted together with cell viability, phenotype preservation, genomic safety, and functional outcome.

### 4.2. Stability and Heterogeneity of Engineered MSC Populations

Engineering primary MSCs introduces variability beyond the intrinsic heterogeneity already associated with donor characteristics, tissue source, culture conditions, and passage history [8]. Individual cells within an engineered population may differ in editing status, transgene copy number, dCas9-effector abundance, sgRNA expression, or responsiveness of the targeted locus. As a result, similar bulk editing percentages can mask substantial differences in the distribution and functional behaviour of individual cells. Stable CRISPRa systems introduce an additional concern related to persistence and epigenetic silencing of the regulatory cassette. Integration of a dCas9-activator system can support prolonged expression, but stable integration does not necessarily guarantee sustained functional activity. Studies in human pluripotent stem-cell systems have shown that dCas9-VPR expression can decline during differentiation in association with promoter hypermethylation, with partial recovery following pharmacological demethylation [51]. Although these observations were not generated in primary therapeutic MSC manufacturing, they demonstrate that activator expression can depend on chromatin context and that long-term CRISPRa function should be verified rather than assumed.

Population heterogeneity is particularly relevant for CRISPRa because transcriptional response can vary according to activator abundance, sgRNA expression, chromatin accessibility, and cellular state [8,52]. Single-cell cloning can enrich highly responsive cells, but prolonged clonal expansion may alter proliferative capacity, differentiation potential, or secretory function. For therapeutic MSC products, extensive clonal selection may therefore create a more molecularly uniform population while simultaneously reducing its biological representativeness or altering important MSC characteristics. Culture history may contribute additional variability. Mechanical-memory studies have demonstrated that substrate stiffness can influence lineage-associated epigenetic states in MSCs and that some effects may persist after the cells are transferred to a different environment [53,54,55,56]. These observations should not be interpreted as established release requirements for CRISPR-engineered MSCs, but they illustrate how culture-associated variables can potentially influence chromatin state, responsiveness to transcriptional regulation, and phenotypic stability. Accordingly, characterization of engineered MSC populations should extend beyond confirmation of an average editing or expression level. Longitudinal evaluation of engineering stability, cellular identity, senescence, proliferative capacity, and functional output is particularly important when cells undergo prolonged expansion after genetic modification.

### 4.3. Manufacturing Scalability and Population Consistency

Clinical translation requires engineering strategies that can be implemented reproducibly at manufacturing scale. Approaches based on extensive single-cell cloning or prolonged selection may be useful during platform development but can become impractical when large therapeutic cell numbers are required. Self-selection strategies, optimized sgRNA architectures, transposon-based systems, and non-integrating delivery approaches have therefore been explored as alternatives to labour-intensive clonal workflows [23,28]. For nuclease-mediated editing, transient RNP delivery offers an attractive manufacturing strategy because the editor is introduced in a defined molecular form and is cleared after a relatively short period [5]. Inducible Cas9 systems provide another approach for limiting the duration of nuclease exposure and reducing background editing [57]. CRISPRa presents a different challenge because sufficient expression of the activator system must be maintained for the desired duration without creating unnecessary genomic or manufacturing burden.

Starting-tissue selection is a major process variable. Bone marrow-derived MSCs have a well-established clinical manufacturing history but can show donor- and age-related variation in cell yield and expansion. Adipose-derived MSCs may offer comparatively accessible starting material and high initial stromal-cell yields, whereas umbilical cord-derived MSCs provide a perinatal allogeneic source with favourable expansion potential. These general properties do not establish a universal ranking for CRISPR delivery or editing: efficiency depends on donor, passage, cell-cycle state, nuclease cargo, and delivery conditions. Indeed, the different knock-in efficiencies reported for bone marrow-, adipose-, and umbilical cord blood-derived human MSCs under a related Cas9/AAV6 workflow demonstrate why each source requires process-specific validation [7,8,58].

An illustrative manufacturing workflow includes starting-material procurement and isolation, primary culture and expansion, engineering by plasmid, mRNA, RNP, or vector delivery, optional enrichment/selection, post-engineering expansion, and final identity, purity, viability, potency, sterility, and genomic-safety testing. A published non-engineered bone-marrow MSC GMP process used approximately 28 days for its culture/manufacturing phase, producing bags containing approximately 80–190 million cells; this is an example rather than a validated manufacturing timeline for CRISPR-engineered MSCs [59]. Adding genome modification, selection, clonal evaluation when justified, and extended QC can increase the interval beyond such baseline processes, and the precise schedule should be established for each product. Culture timing should not be confused with total batch-release lead time because the required QC assays can continue after harvest.

Economic feasibility likewise remains product- and scale-dependent. In the same academic study, illustrative direct manufacturing costs were approximately EUR 2633 per clinical-grade bag versus EUR 6647 per GMP-grade bag, excluding some facility, validation, and environmental-monitoring costs [59]. These figures describe conventional MSC production, not a validated cost per dose of CRISPR-engineered MSCs; adding engineering reagents, vector or RNP manufacture, extended process development, genomic characterization, potency testing, and possible cell losses from selection is expected to increase costs. Therefore, no generalized CRISPR-engineered MSC cost per dose should be inferred without a product-specific cost-of-goods model.

Population consistency should also be assessed in relation to MSC identity and therapeutic function. Donor age, tissue source, passage number, and culture conditions can alter proliferation, differentiation, and secretory profiles even before engineering is introduced [8,58]. Genetic modification adds another source of variation, making it important to determine not only what proportion of cells carry the intended modification but also whether edited and unedited subpopulations differ in clinically relevant behaviour. Future manufacturing strategies may therefore benefit from more detailed population-level characterization. Single-cell transcriptomic, chromatin-accessibility, or surface-protein profiling may help identify subpopulations with incomplete engineering, senescence, or unintended differentiation. At present, however, such technologies are more appropriately considered development and characterization tools than routine release assays. Master-cell-bank strategies may improve starting-material consistency when stable engineering is appropriate, while synthetic or logic-gated gene circuits represent future approaches for producing more context-dependent therapeutic responses [23,60]. Ultimately, scalable manufacturing should minimize unnecessary manipulation while preserving viability, expansion capacity, MSC identity, genomic integrity, and the intended functional phenotype. Standardization should therefore be based on reproducible product performance rather than molecular modification alone.

### 4.4. Translational Priorities for Delivery and Manufacturing

Future development of CRISPR-engineered MSC platforms should integrate delivery performance with the requirements of the final therapeutic product. Non-integrating delivery remains particularly attractive for transient nuclease editing and should be further optimized for larger transcriptional-regulatory cargos. At the same time, stable platforms may remain appropriate when persistent expression is mechanistically necessary, if integration, expression stability, and long-term cellular behaviour are adequately characterized. Delivery efficiency should therefore be treated as a process attribute rather than an isolated measure of success. The clinically relevant question is whether the selected delivery system produces a sufficiently homogeneous, viable, stable, and functionally potent MSC population [61]. Tissue source, passage number, expansion requirements, duration of the intended therapeutic effect, and route of administration should all be considered when selecting the engineering platform. Cell-tracking and biodistribution approaches may provide useful additional information during preclinical development, although their role should remain indication- and product-specific [49,62]. These considerations shift the development objective from maximizing editing efficiency to establishing a controlled manufacturing process in which molecular engineering can be linked reproducibly to the final therapeutic phenotype.

## 5. Safety, Immunogenicity, and Long-Term Stability

The introduction of programmable genome or transcriptional engineering creates safety considerations that extend beyond the intrinsic risks of conventional MSC products. These include unintended genomic modification, immune recognition of editing components, phenotypic instability, abnormal persistence, and changes in pathways governing proliferation or senescence. Importantly, these risks differ between engineering modalities and should therefore be assessed according to the specific architecture and intended mechanism of the final MSC product.

### 5.1. Off-Target Editing and Genomic Integrity

Off-target activity occurs when a CRISPR nuclease cleaves genomic sites other than the intended locus. Potential consequences include small insertions or deletions, larger structural alterations, and activation of DNA-damage responses. The biological significance of these events depends on the affected genomic region, frequency of the alteration, and whether cells carrying the change acquire a selective advantage during subsequent expansion [44,63]. This issue is particularly relevant for MSC products because gene editing may be followed by substantial ex vivo expansion. Rare genomic events that initially represent a minor fraction of the population can become more important if they influence proliferation, senescence, or survival. The LATE assay, for example, identified low-frequency growth-promoting events in primary human MSCs that were not adequately predicted by computational guide analysis alone [64]. These findings support the use of empirical genomic and functional assessment in the relevant cellular context rather than relying exclusively on in silico guide prediction.

Several complementary approaches can improve editing precision. High-fidelity Cas9 variants, including eSpCas9, SpCas9-HF1, and HiFi Cas9, are designed to reduce non-specific DNA interactions while retaining useful on-target activity [22]. Chemically modified sgRNAs may improve guide stability and increase the ratio of on-target to off-target activity in RNP-based workflows [65]. Paired nickases and inducible nuclease systems can provide additional control over cleavage [57]. Empirical detection methods such as GUIDE-seq, DISCOVER-seq, and related genome-wide or targeted assays can provide additional information regarding unintended cleavage [63]. No single method captures every category of genomic alteration, and safety assessment should therefore combine guide prediction with experimentally appropriate genomic analyses. When engineered MSCs undergo prolonged post-editing expansion, genomic results should also be interpreted alongside cell growth, senescence, and functional data. The objective is not merely to demonstrate low off-target frequency at the time of editing, but to establish that the final expanded MSC population remains genetically and biologically stable.

### 5.2. Immunogenicity and Immune-Evasion Engineering

Immune recognition of CRISPR-engineered MSCs can arise from several distinct sources. Bacterial Cas proteins such as SpCas9 may be recognized by pre-existing antibodies or T cells in some individuals [4]. Viral-vector components may provide additional innate or adaptive immune stimuli when viral delivery is used. Furthermore, allogeneic MSCs should not be assumed to be completely immune privileged, as inflammatory signals can alter HLA expression and influence interactions with recipient immune cells [36,66]. Immune-evasion engineering has therefore been investigated as a strategy for reducing allogeneic recognition. Disruption of B2M can reduce expression of classical HLA class I molecules, whereas modification of CIITA can reduce inducible HLA class II expression [31,36]. However, a reduction in classical MHC class I expression introduces an important biological trade-off because cells with low MHC class I expression may become more susceptible to natural killer (NK)-cell-mediated “missing-self” recognition [31].

Additional expression of non-classical inhibitory HLA molecules such as HLA-E or HLA-G has been explored as one potential approach to limiting NK-cell recognition [39]. Such multi-gene strategies may improve immune compatibility but substantially increase engineering complexity. Their effects should therefore be evaluated using functional T-cell and NK-cell assays rather than relying exclusively on altered expression of individual surface markers. Immunogenicity can also be influenced by the duration of editor exposure. Transient RNP or mRNA delivery reduces persistence of intracellular Cas protein compared with stable expression systems, while reduced-immunogenicity nuclease variants or epitope-engineered Cas proteins may provide additional strategies for decreasing immune recognition [43,67]. Importantly, immune-evasion engineering should remain conceptually distinct from enhancement in MSC immunomodulatory potency. Reducing recognition of an allogeneic MSC does not necessarily increase its therapeutic immunosuppressive activity, and vice versa. Each modification should therefore be characterized for both its intended immunological effect and its potential impact on MSC identity, viability, and function.

### 5.3. Persistence, Senescence, and Long-Term Safety

The desired persistence of an engineered MSC product is dependent on its intended therapeutic mechanism. In many regenerative applications, therapeutic benefit may result from transient paracrine signalling, immune modulation, or interaction with injured tissues rather than permanent engraftment. Longer cellular persistence should therefore not automatically be considered advantageous [32]. This issue is particularly important when engineering pathways that enhance survival. Increased resistance to apoptosis may extend the duration of MSC activity, but prolonged survival can also increase exposure to unintended effects if the cells acquire an abnormal phenotype. In oncologic settings, persistent MSCs may present additional concerns because MSC-derived factors can exert context-dependent pro-angiogenic or immunosuppressive effects [68].

Tissue source, passage number, culture history, and post-editing selection can also influence the persistence of the engineered phenotype [8,69]. Cellular senescence or epigenetic drift may reduce functional activity even when cells remain detectable, whereas selective expansion of rare cell populations may produce the opposite problem by enriching cells with altered growth properties [44,56]. Persistence studies should therefore distinguish between persistence of the cell and persistence of the intended therapeutic function. Additional safety mechanisms may be considered when persistent, engineered cells or integrated constructs are used. Inducible kill-switch systems, including inducible caspase-9 or herpes simplex virus thymidine kinase, provide a potential mechanism for eliminating administered cells if severe adverse behaviour occurs [70]. Inducible expression systems can provide temporal control over engineered functions, while safe-harbour targeting and chromatin-insulating elements may reduce some position-dependent effects when stable integration is required [71,72]. Particular caution is warranted when editing pathways involved in senescence or proliferative lifespan. CRISPRa/i interrogation of the CDKN2A/CDKN2B regulatory region in human MSCs has demonstrated that non-coding regulatory elements can modulate senescence-associated expression [73]. In a distinct bovine-MSC context, knockout of TP53 or PTEN increased proliferative capacity but was accompanied by reduced differentiation potential [74]. Although these studies involve different species, targets, and experimental objectives, they highlight the need to preserve checkpoint integrity when engineering pathways associated with cell growth. Long-term safety assessments should therefore integrate genomic stability, proliferation, senescence, phenotype, persistence, and mechanism-linked potency rather than relying on any single molecular measurement.

## 6. Potency and Regulatory Translation

The translational development of CRISPR-engineered MSCs requires integration of the regulatory principles applicable to both advanced cell products and genetically modified therapeutic products. The relevant requirements will depend on the engineering strategy, delivery platform, persistence of the modification, intended mechanism of action, and clinical indication. Accordingly, the regulatory focus should be placed on the properties of the final engineered MSC product rather than on the CRISPR platform in isolation.

### 6.1. Regulatory Considerations for CRISPR-Engineered MSCs

Somatic genome engineering of ex vivo-expanded MSCs introduces additional characterization requirements beyond those applied to conventional MSC products. Relevant considerations include the nature of the editing system and delivery vector, editing efficiency and heterogeneity, genomic integrity, off-target activity, persistence of the engineered phenotype, immune consequences of the modification, and the potential impact of engineering on MSC identity and functional activity [4,75,76]. International governance frameworks for human genome editing emphasize oversight, traceability, risk-based assessment, and long-term consideration of somatic genome-editing applications [75]. For cell-based products, these principles must be integrated with established expectations for starting-material control, reproducible manufacturing, product characterization, and demonstration of clinically meaningful biological activity.

The U.S. FDA approval in December 2024 of the allogeneic bone marrow-derived MSC product remestemcel-L-rknd (Ryoncil) for pediatric steroid-refractory acute graft-versus-host disease [77] provides an important example of regulatory evaluation of a culture-expanded allogeneic MSC product. However, remestemcel-L is not a genetically engineered product and therefore should not be interpreted as establishing a direct regulatory pathway for CRISPR-engineered MSCs. Its relevance is instead indirect: it illustrates the importance of manufacturing consistency, donor and starting-material control, product characterization, and demonstration of biological activity in an MSC therapeutic product [58,78].

As of the November 2026 literature and regulatory review, no FDA-approved CRISPR-engineered MSC product was identified. Clinical translation of CRISPR-modified MSCs remains predominantly at the preclinical stage, and trial registration records must be distinguished from evidence of administered CRISPR-engineered MSC products. An observational study involving Kabuki syndrome (NCT03855631) [79] has investigated the potential application of CRISPR-based epigenome editing using patient-derived cellular models; however, this study did not involve the clinical administration of engineered MSCs. In contrast, an early-phase clinical trial (NCT06446050) [80] has been registered to evaluate genetically modified umbilical cord-derived MSCs for advanced colorectal cancer. Nevertheless, the registered study does not specify CRISPR-based engineering, and therefore should not be classified as a clinical trial of CRISPR-engineered MSCs.

Genetic engineering adds further layers of product-specific characterization rather than replacing these core requirements. Developers must demonstrate not only that the intended genetic modification is present, but also that the resulting MSC population retains appropriate identity, stability, safety, and reproducible therapeutic function [43,67,76]. Regulatory development should therefore begin with the final product profile and intended mechanism of action. The engineering method, manufacturing process, genomic characterization, potency testing, and clinical monitoring should be aligned with the biological function that the modified MSC is expected to provide [78,81].

### 6.2. Functional Potency Assessment

Potency testing represents a central challenge for engineered MSC products because confirmation of a molecular edit does not by itself demonstrate therapeutic activity. Editing efficiency, target-gene expression, or protein abundance may provide useful characterization data, but they should not automatically be regarded as equivalent to functional potency [9,61,78]. A mechanism-linked potency strategy should establish a relationship between the intended molecular modification and the biological activity relevant to the therapeutic indication (Figure 5). For example, CXCR4-mediated MSC homing strategies should be evaluated using migration or chemotaxis assays rather than CXCR4 expression alone, irrespective of whether the modification uses CRISPR or an earlier gene-transfer approach [33]. Similarly, MSC engineering intended to improve angiogenesis should be assessed using functional vascular readouts rather than transgene expression alone [35].

For survival-enhancing strategies, functional resistance to relevant cellular stress may provide more meaningful evidence than target-gene expression alone [25,32]. Likewise, engineering intended to strengthen immunomodulatory activity should be supported by assays that measure the relevant interaction with immune cells rather than by measurement of a single immunomodulatory marker. A rational potency framework can therefore be conceptualized as a progression from confirmation of the molecular modification to verification of its downstream biological effect, to demonstration of a mechanism-linked functional response. The final potency assay should reflect the intended mechanism of action and be sufficiently reproducible to support comparability and manufacturing control. This principle is particularly important for CRISPR-engineered MSCs because highly efficient editing does not guarantee an equivalent therapeutic effect. Conversely, a heterogeneous engineered population may retain adequate potency if the relevant biological function is consistently maintained. Potency should therefore provide the bridge between molecular engineering and the functional quality of the final cell product.

### 6.3. Secretome and Extracellular Vesicle Considerations

If genetic engineering is used to alter the secretome or extracellular vesicle output of MSCs, potency assessment warrants additional consideration. Although engineering of the parental MSC may alter the quantity or composition of secreted cytokines, proteins, nucleic acids or extracellular vesicles, the magnitude and functional relevance of such alterations cannot be predicted based on the intracellular genetic modification alone. MISEV2023 offers a contemporary framework for characterization and reporting of extracellular vesicles, including particle-, biochemical-, and marker-related attributes [82]. These parameters are important in defining EV preparations but do not in and of themselves establish therapeutic potency.

Studies on MSC-derived EVs show the need to separate characterization and functional activity. Preparations with apparently similar physical or biochemical characteristics may have different biological effects, and functional assays such as immune-cell proliferation or mixed-lymphocyte responses may provide more mechanism-relevant information for immunomodulatory applications [83,84,85,86]. Therefore, if CRISPR engineering is used to improve a therapeutic mechanism mediated by the secretome or EVs, product development should demonstrate a link between the modification of the cells, the resultant secreted output, and the relevant biological function. Expression of the engineered target alone should not be taken as evidence of potency. No universal potency assay for all MSC-derived EV applications exists and therefore assay selection should remain product- and mechanism-specific [86,87].

## 7. Applications for Specific Diseases

CRISPR-engineered MSCs have been explored across cardiovascular, musculoskeletal, inflammatory, neurological, and oncologic models. Most of the current evidence remains preclinical, and the principal value of these studies is therefore to demonstrate how specific engineering strategies can be matched to disease-associated biological limitations. Different indications require different functional outputs; consequently, the same editing strategy should not be assumed to provide equivalent therapeutic benefit across diseases [2,7,25,32,40]. Broader therapeutic uses of conventional MSCs have been reviewed separately [88].

### 7.1. Cardiovascular and Ischemic Diseases

Cardiovascular and ischemic models illustrate the potential value of engineering multiple complementary MSC functions. The ischemic microenvironment exposes administered cells to hypoxia, oxidative stress, and nutrient deprivation, making cell survival a major limitation. In human bone marrow-derived MSCs, CRISPR/Cas9-mediated KEAP1 knockout enhanced NRF2-related oxidative stress protection and improved perfusion in a mouse limb-ischemia model [32]. Separately, CRISPRa-mediated PDGFR-β activation in adipose-derived MSCs improved survival, paracrine activity, and healing in diabetic-wound models [25]. Homing represents another important target. CXCR4 overexpression has improved MSC trafficking to infarcted myocardium in a rat study, although this used retroviral transfer rather than CRISPR editing [33]. Non-CRISPR E-selectin modification of MSCs has likewise improved vascularization and repair in an ischemic-wound model, illustrating an alternative proangiogenic engineering approach [35]. These approaches illustrate that survival, homing, and angiogenic activity represent distinct but interconnected therapeutic functions. A highly persistent MSC may still provide limited benefit if it does not reach the relevant tissue, while efficient homing may be insufficient if the cell does not deliver an appropriate paracrine or angiogenic response. Future studies should therefore determine whether simultaneous modification of these complementary functions provides additional benefit without compromising safety or manufacturing consistency.

### 7.2. Musculoskeletal and Cartilage Repair

Musculoskeletal applications place greater emphasis on lineage regulation, matrix formation, and control of undesirable differentiation. CRISPR/Cas9-mediated RUNX2 knockout has been studied in engineered cartilage matrices [30], while dCas9-mediated SOX9 activation combined with PPARγ repression in rat bone marrow-derived MSCs enhanced chondrogenesis and calvarial bone regeneration [40]. Editing of RUNX2 and VEGF in MSC-derived engineered extracellular matrices has also been explored to modify hypertrophy, vascularization, and ectopic ossification in osteochondral repair models [30]. These findings illustrate how genome engineering can influence not only the fate of the MSC but also the composition and biological properties of the matrix generated by the engineered cells. Inflammatory control may provide an additional therapeutic component in degenerative musculoskeletal disease. CRISPRa-mediated TSG-6 activation altered the protein cargo of EVs from an immortalized human adipose-derived MSC line and modulated inflammatory-marker expression in an in vitro human intervertebral-disc cell model; the engineered EVs did not demonstrate superior efficacy over control EVs [26]. For musculoskeletal applications, engineering should therefore be evaluated using tissue-relevant functional endpoints that integrate differentiation, matrix quality, inflammation, and inappropriate tissue formation rather than relying exclusively on expression of lineage-associated genes.

### 7.3. Inflammatory and Immune-Mediated Diseases

MSC therapies are frequently investigated for their ability to regulate inflammatory and immune responses. CRISPR-based engineering provides an opportunity to strengthen these functions by modifying endogenous immunomodulatory pathways, including TSG-6, IDO-associated mechanisms, and related regulatory signals [2,26]. A separate but related objective is the reduction in immune recognition in allogeneic MSC products. Engineering of antigen-presentation pathways involving B2M or CIITA has been investigated to reduce T-cell recognition, while additional modulation of non-classical HLA molecules may be required to address NK-cell responses [31,36,39]. These two strategies should remain conceptually distinct. Increasing the immunomodulatory activity of an MSC is intended to strengthen its therapeutic influence on the recipient immune system, whereas immune-evasion engineering is intended to reduce elimination of the administered MSC itself. A product may require one or both properties depending on the indication and manufacturing model. Future development in inflammatory or immune-mediated disease should therefore define whether the primary mechanism of action is direct immunomodulation, prolonged persistence of an allogeneic cell product, or a combination of both. Potency assays should then be aligned with the corresponding mechanism rather than using general immune-marker expression as a surrogate for efficacy.

### 7.4. Neurological and Oncological Diseases

Neurological applications of MSCs are strongly associated with paracrine and extracellular-vesicle-mediated mechanisms. MSC-derived secreted factors and EVs have been investigated for their ability to influence oxidative stress, inflammation, and neurotrophic signalling, including pathways associated with Nrf2/HO-1 activity [62,69,89]. CRISPR-based engineering could potentially strengthen these functions by increasing selected neuroprotective or anti-inflammatory outputs, although evidence for engineered primary MSC products in neurological indications remains relatively limited.

Oncologic applications involve a substantially different therapeutic concept. MSCs possess tumour-associated migratory properties that have prompted investigation of engineered cells as delivery vehicles for anti-tumour factors. For example, a CXCL10- and Nrf2/FTH-associated engineered MSC platform demonstrated immunological effects in a preclinical glioblastoma model [49]. However, oncologic applications require particular caution because MSCs can exert context-dependent effects within the tumour microenvironment. Their secretory and immunomodulatory functions may influence angiogenesis, inflammation, and immune-cell behaviour in ways that are not universally anti-tumour [68]. Engineering strategies for cancer should therefore demonstrate that the intended anti-tumour mechanism outweighs any unintended trophic, angiogenic, or immunosuppressive effects. Together, disease-specific studies emphasize that CRISPR engineering should not be developed as a generic method for increasing MSC activity. The engineering target, desired phenotype, potency assay, and safety strategy should be selected according to the biological limitations and therapeutic requirements of the intended indication. Representative disease-oriented studies are summarized in Table 2 to distinguish the origin of the engineered cells, the genome regulation strategy, the delivery method, the experimental model, and the reported functional endpoint. These examples involve ex vivo engineered cells or engineered-cell-derived products evaluated in preclinical or in vitro systems; none establishes clinical efficacy of a CRISPR-engineered MSC product.

## 8. Future Directions and Emerging Technologies

The continuous development of CRISPR technologies is pushing the limits of MSC engineering beyond the traditional Cas9-mediated DSB formation and the first-generation transcriptional activation. Emerging platforms are increasingly focusing on precise sequence modification, non-cleaving gene regulation, programmable epigenetic control and improved temporal control of engineered functions. These approaches may overcome some limitations of conventional editing, although their use in primary therapeutic MSCs is less mature.

### 8.1. Base and Prime Editing Beyond Double-Strand Breaks

Base editing and prime editing provide alternative strategies for precise genomic modification without relying on conventional Cas9-induced DSBs. Base editors combine a Cas-derived DNA-targeting module with a nucleotide-modifying enzyme, enabling selected base conversions within a defined editing window. Prime editors combine a Cas9 nickase with reverse-transcriptase activity and a prime-editing guide RNA to introduce a broader range of small sequence substitutions, insertions, or deletions [90,91]. These approaches may be particularly relevant to primary MSCs because they reduce dependence on HDR, which is less efficient in slowly proliferating cells. Potential applications include correction of defined sequence variants, modification of regulatory loci, or introduction of precise functional changes without generating a conventional DSB. However, the available evidence in primary therapeutic MSCs remains considerably less developed than that for conventional Cas9-mediated knockout. The results obtained in pluripotent stem cells or other model systems should therefore not be assumed to translate directly to MSC manufacturing [73,92].

DSB-independent editing also does not eliminate the need for specificity assessment. Base editing can produce bystander nucleotide changes within the editing window, while prime editing can generate heterogeneous or unintended sequence outcomes depending on target and guide design [91]. These platforms will consequently require product-specific characterization of editing accuracy, genomic consequences, and functional outcome before their advantages can be translated into MSC therapeutics.

### 8.2. Epigenome Editing and Next-Generation Gene Regulation

Future MSC engineering is also likely to expand beyond conventional gene activation toward more sophisticated control of endogenous regulatory networks. dCas9 can be coupled to transcriptional repressors, chromatin-modifying enzymes, or other regulatory domains to alter gene activity without directly cleaving DNA [6,24]. Such platforms include CRISPR interference (CRISPRi), locus-specific epigenome editing, and programmable modulation of regulatory elements. The potential advantage of these approaches lies in the ability to modify pathway activity without permanently disrupting the underlying genomic sequence. Human MSC studies using CRISPRa/i to interrogate regulatory regions at the CDKN2A/CDKN2B locus demonstrate that non-coding regulatory elements can be manipulated to alter senescence-associated transcription [73]. This provides a conceptual basis for engineering cellular behaviour through regulatory elements rather than coding-sequence disruption. The duration of such regulation will depend strongly on the delivery strategy. A transiently delivered regulator may produce temporary gene modulation, whereas stable vector-based expression can generate a more persistent state. Consequently, claims of “reversible” CRISPR regulation should be interpreted in the context of the complete delivery architecture rather than the dCas9 system alone.

Future development may also involve multiplex regulation, in which several endogenous pathways are modified simultaneously. This approach could be particularly relevant to MSCs because therapeutic function is rarely determined by a single gene. Survival, migration, immunomodulation, paracrine activity, and differentiation are interconnected, and coordinated regulation may provide more physiologically relevant enhancement than maximization of one isolated pathway. However, increasing engineering complexity also increases the requirements for characterization. Multiplex regulatory systems must be assessed not only for molecular activity but also for interactions between targeted pathways, population heterogeneity, stability, delivery burden, and downstream therapeutic potency. Future progress in CRISPR-based MSC engineering is therefore likely to shift from maximizing molecular editing efficiency toward designing controllable and indication-specific engineering systems. The most clinically relevant platforms will be those in which the molecular modification, functional potency, manufacturing consistency, and safety profile can be evaluated as an integrated product rather than as independent technical achievements.

## 9. Conclusions

CRISPR-based engineering provides complementary strategies for modifying MSC survival, migration, paracrine activity, immunomodulatory properties, and lineage-associated functions. Nuclease-mediated genome editing is particularly suited to applications requiring permanent gene disruption or targeted sequence modification, whereas dCas9-based transcriptional regulation enables modulation of endogenous gene expression without permanent alteration in the underlying DNA sequence. Emerging base-, prime-, and epigenome-editing technologies may further expand the range and precision of MSC engineering. Despite these advances, current evidence remains predominantly preclinical, and several barriers continue to limit clinical translation, including efficient delivery, population heterogeneity, genomic and phenotypic stability, immunogenicity, manufacturing consistency, and the establishment of mechanism-linked potency assays. Future development should therefore focus on matching the engineering modality to a clearly defined therapeutic mechanism while demonstrating that the resulting MSC product can be manufactured reproducibly and maintains appropriate safety, stability, and functional potency. Ultimately, the translational value of CRISPR-engineered MSCs will depend not on molecular modification alone, but on whether that modification produces a consistent and clinically meaningful therapeutic function.

## Figures and Tables

**Figure 1:**
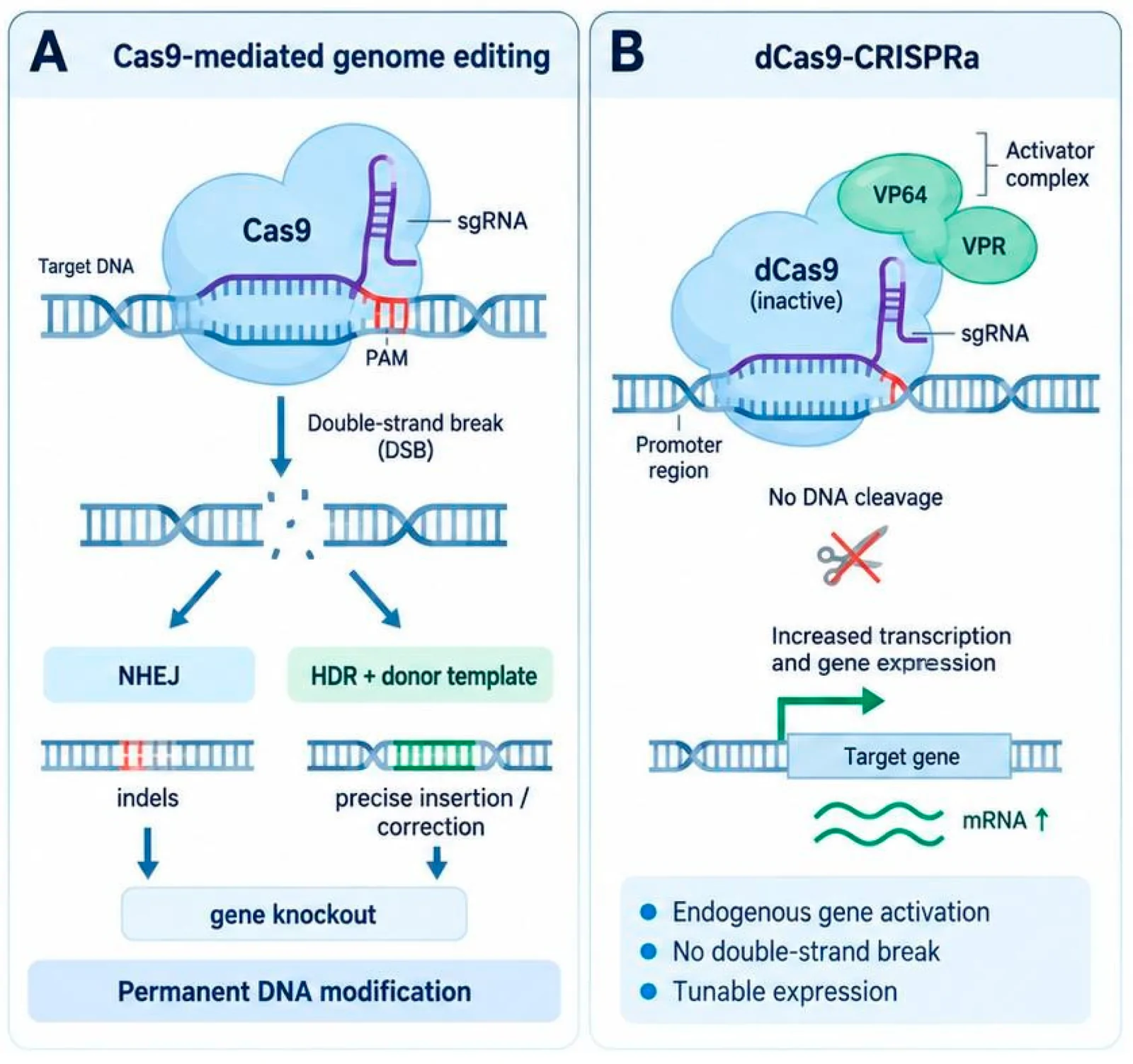
CRISPR-based strategies for MSC engineering. (**A**) Cas9-mediated genome editing generates a double-strand DNA break (DSB), which can be repaired through non-homologous end joining (NHEJ), resulting in insertions or deletions that may disrupt gene function, or through homology-directed repair (HDR) in the presence of a donor template, enabling precise sequence modification. (**B**) Nuclease-dead Cas9 (dCas9) coupled to transcriptional activators enables CRISPR activation (CRISPRa), increasing transcription of endogenous target genes without inducing a DSB.

**Figure 2:**
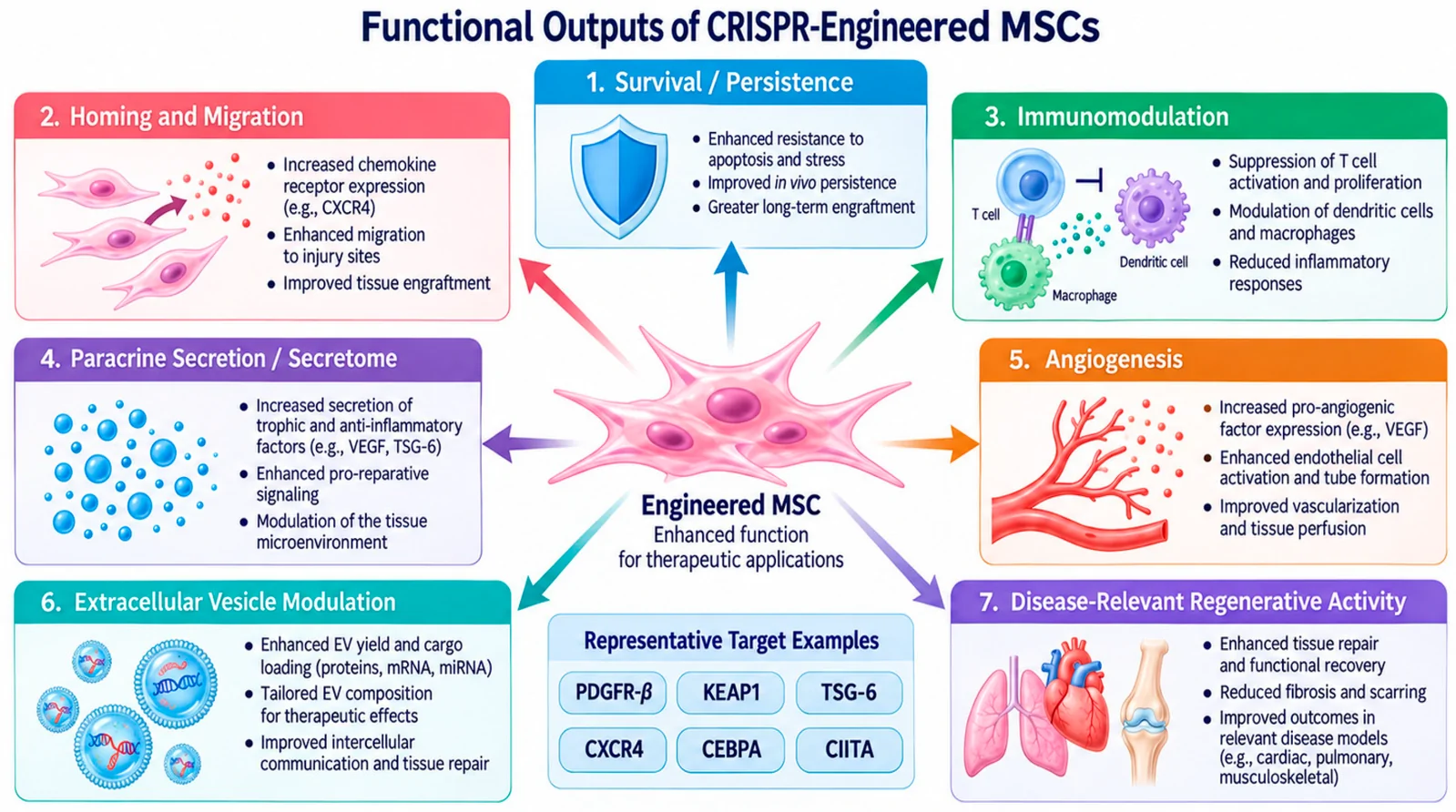
Functional outputs of CRISPR-engineered MSCs. CRISPR-based strategies can modify MSC survival and stress resistance, homing and tissue retention, paracrine and extracellular vesicle activity, immunomodulatory and immune-compatibility properties, and lineage- or matrix-associated functions. Gene labels within the schematic represent illustrative target pathways and should not be interpreted as evidence that every target has been validated by CRISPR engineering in MSCs.

**Figure 3:**
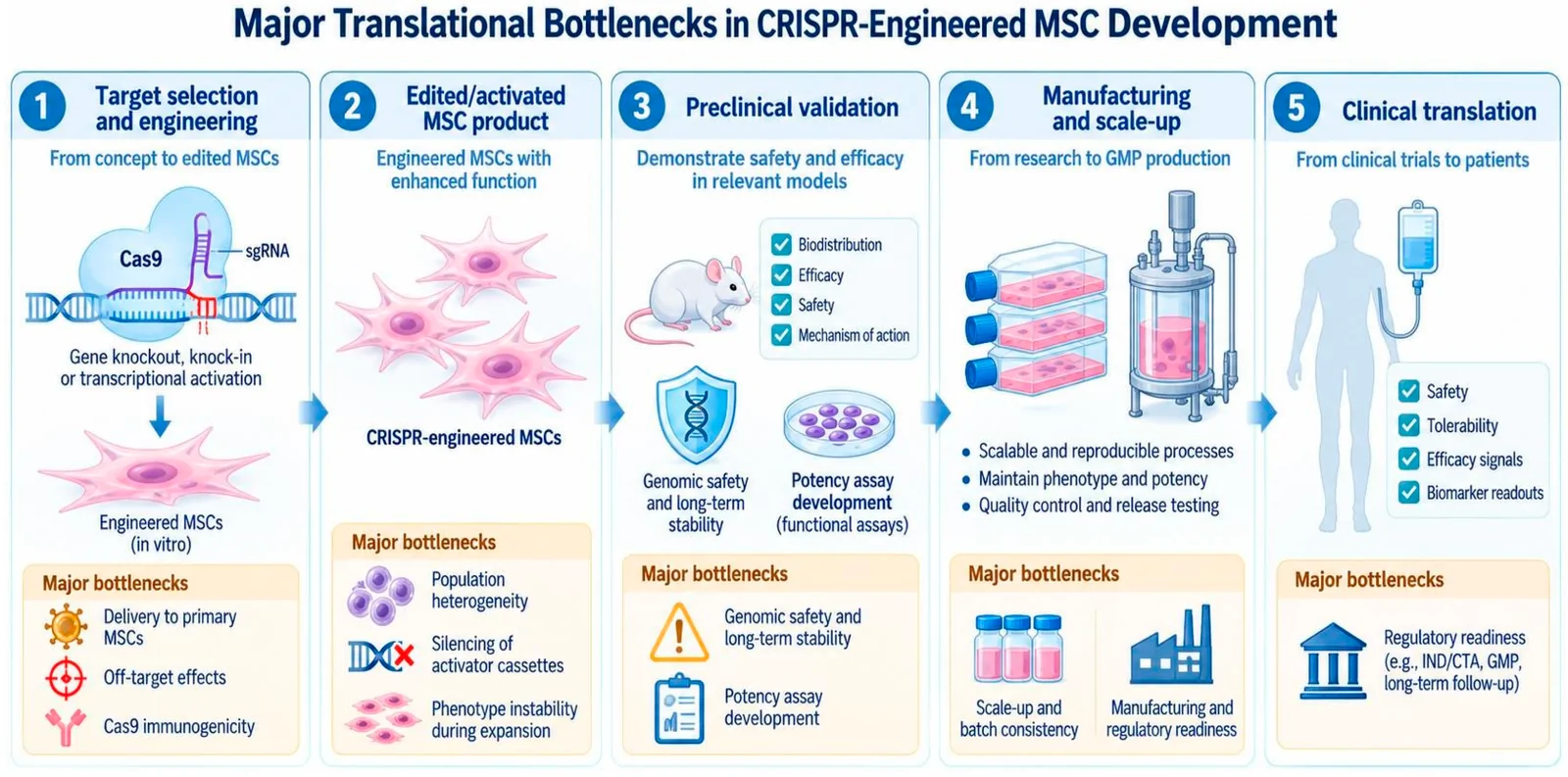
Major translational bottlenecks in CRISPR-engineered MSCs development. Successful clinical translation depends on product consistency, safety, scalable manufacturing, and measurable potency, rather than editing efficiency alone.

**Figure 4:**
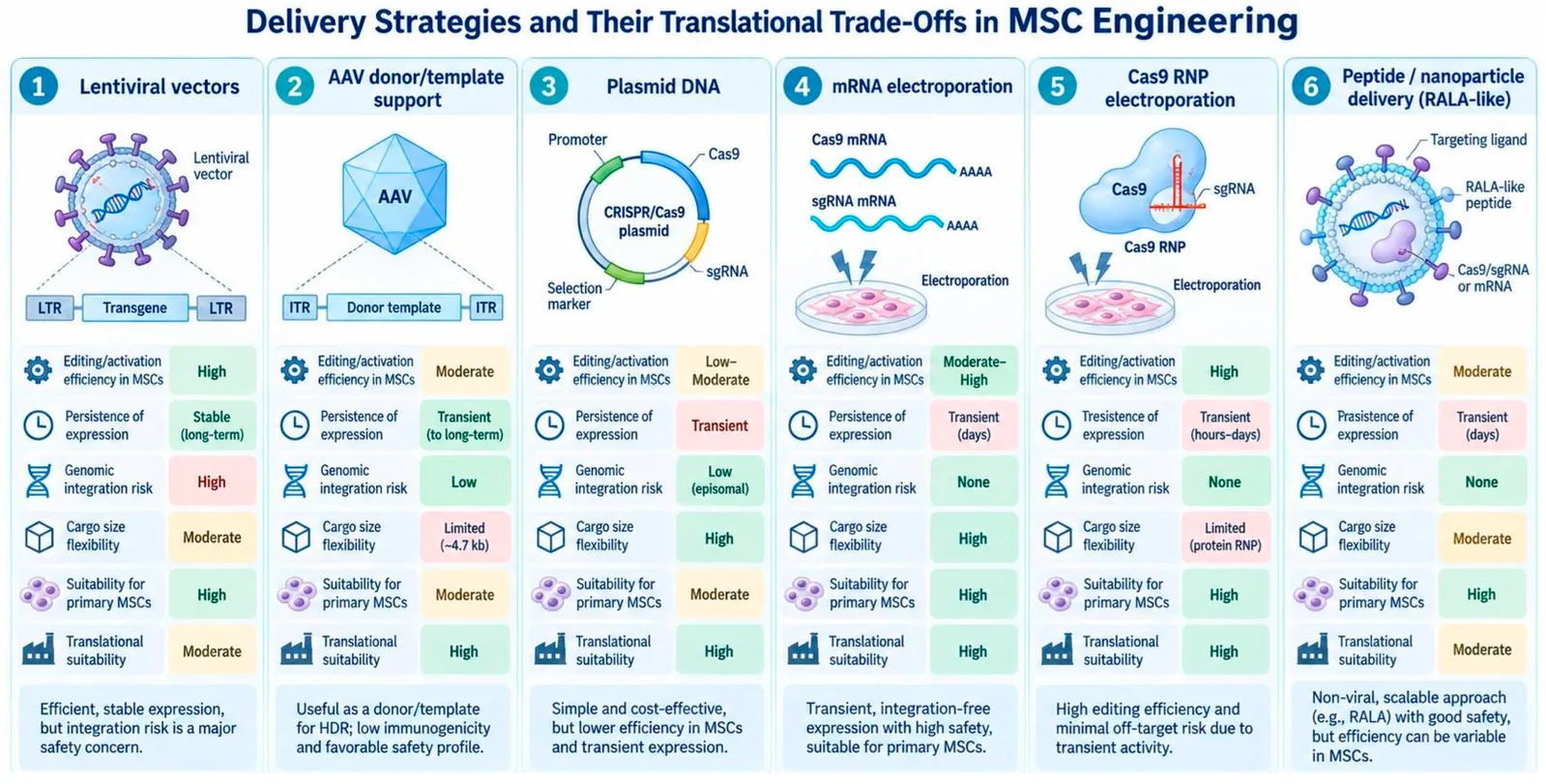
Delivery strategies and their translational trade-offs in MSC engineering. The illustrated platforms primarily represent in vitro/ex vivo engineering of MSCs before administration, rather than direct in vivo delivery. Viral and non-viral systems differ in efficiency, persistence, integration risk, cargo flexibility, and suitability for primary MSCs. Approximate capacities and practical cargo constraints are discussed in Section 4.1.

**Figure 5:**
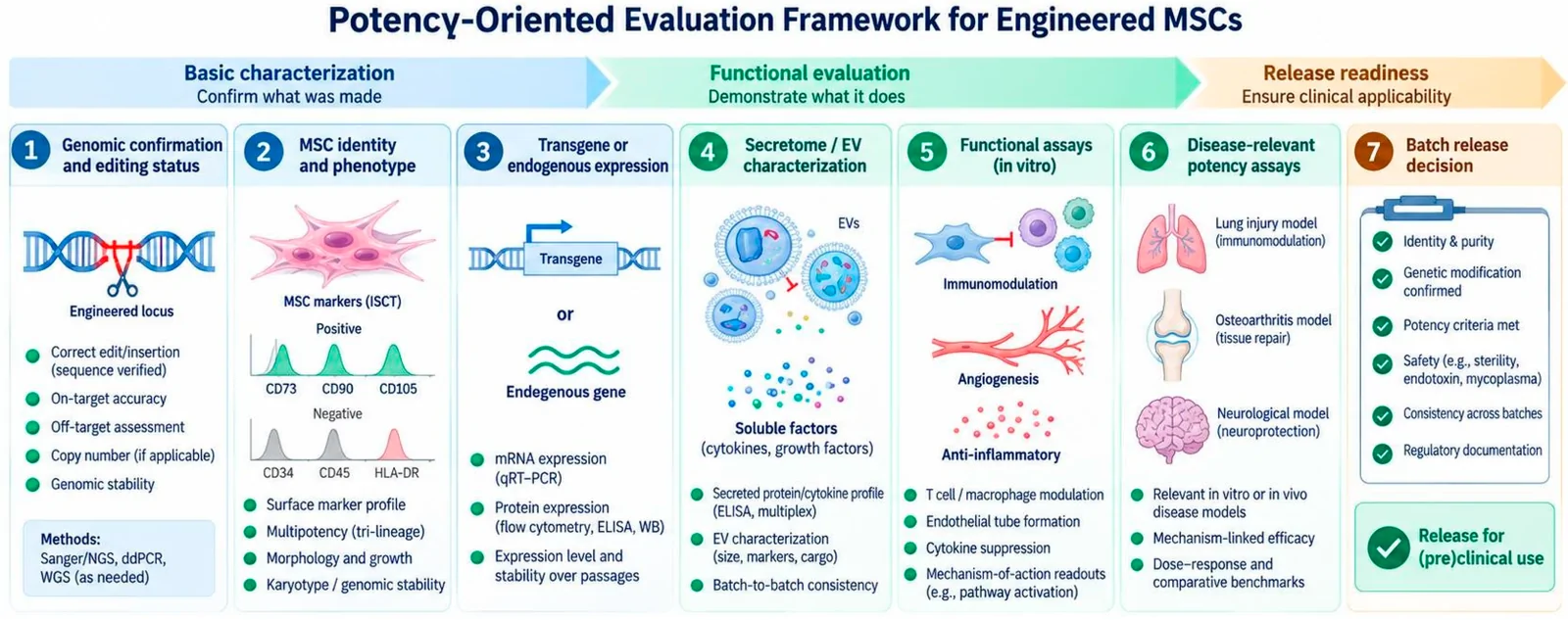
Conceptual potency-oriented evaluation framework for CRISPR-engineered mesenchymal stem cells (MSCs). Evaluation progresses from confirmation of the intended genomic modification and preservation of MSC identity to verification of target expression and mechanism-relevant functional activity. Secretome or extracellular vesicle characterization may be included when relevant to the proposed mechanism of action. Potency assessment should link the engineered molecular change to a therapeutically relevant biological function, while product-release decisions integrate identity, purity, safety, manufacturing consistency, and predefined potency criteria. The specific assays and acceptance criteria should be adapted to the engineering strategy, mechanism of action, and intended clinical application.

**Table 1:** Representative CRISPR-based strategies for functional engineering of MSCs.

Therapeutic Objective/Target	MSC Species and Tissue Source	CRISPR-Engineering Strategy	Reported Functional Outcome	Ref.
Targeted insertion/repair	Human primary MSCs: bone marrow, adipose tissue, and umbilical cord blood	Cas9 RNP with AAV6 donor template (HDR)	Locus- and source-dependent targeted integration; engineered human MSCs improved wound healing in diabetic mice	[7]
Inflammatory modulation/TSG-6	Human adipose-derived immortalized MSC line (ASC52telo)	dCas9-mediated transcriptional activation	Increased TSG-6 in MSC-derived EVs and reduced inflammatory-marker expression in an IL-1β-stimulated human IVD cell assay; superiority over control EVs was not demonstrated	[26]
Transcriptional regulation/CEBPA, CIITA	Human primary bone marrow-derived MSCs	Lentiviral CRISPR/SAM activation	Induced target-gene transcription and downstream phenotype including HLA-DR expression	[27]
Cartilage matrix/VEGF, RUNX2	Human bone marrow-related MSC line (MSOD-B), not freshly isolated donor MSCs	Cas9-mediated knockout of VEGF or RUNX2	Altered extracellular matrix properties; RUNX2 knockout reduced hypertrophy and improved cartilage-repair readouts	[30]
Immune evasion/B2M, HLA-E, HLA-G	Human embryonic-stem-cell-derived MSCs (not tissue-isolated adult MSCs)	B2M knockout with HLA-E/HLA-G transgene expression	Enhanced resistance to several natural-killer-cell subtypes in experimental models	[31]

**Table 2:** Disease-related experimental models, CRISPR strategies, delivery methods, and measured functional endpoints for engineered MSC platforms.

Disease/Model	MSC Origin/Product	CRISPR Modality and Target	Delivery Strategy	Functional Endpoint/Evidence
Chronic limb-threatening ischemia (mouse hindlimb ischemia model)	Human bone marrow-derived MSCs	CRISPR/Cas9-mediated KEAP1 knockout (NRF2 pathway activation)	Cas9–sgRNA RNP nucleofection (4D-Nucleofector)	Reduced oxidative stress and increased MSC viability and proangiogenic signaling; improved tissue perfusion and arteriogenesis in mice; preclinical [32]
Diabetic cutaneous wound healing (db/db mouse)	Human BM-, adipose-, or cord-blood-derived MSCs	Cas9 RNP + AAV6 donor; integration of PDGFB or VEGFA expression cassettes	RNP electroporation and AAV6 donor-vector delivery	Accelerated wound closure vs. unmodified MSCs in mouse model; preclinical [7]
Osteochondral defect and cartilage matrix (rat)	Human MSC-derived MSOD-B line; devitalized cartilage matrices	Cas9-mediated VEGF or RUNX2 knockout	Cas9/gRNA plasmid transfection; clone selection	RUNX2-modified matrices reduced hypertrophy and improved cartilage-repair outcomes; preclinical [30]
Inflammatory intervertebral-disc cell model (human in vitro)	Human immortalized adipose-derived MSC line (ASC52telo); EV product	dCas9/SAM activation of TSG-6	Multi-component lentiviral CRISPRa system	TSG-6-rich EVs altered cargo and attenuated inflammatory-marker expression; no clear superiority over control EVs [26]
Natural-killer-cell recognition/immune-evasion assays	Human embryonic-stem-cell-derived MSCs (EMSCs)	B2M knockout in hESCs, followed by HLA-E/HLA-G expression	Genome editing before MSC differentiation; lentiviral transgene delivery	Co-expression reduced killing by multiple NK subtypes; in vitro and mouse xenograft evidence [31]

Note: BM, bone marrow; EV, extracellular vesicle; IVD, intervertebral disc; NK, natural killer; RNP, ribonucleoprotein. Model species and engineered-cell species are indicated separately.

## Data Availability

No new data were created or analyzed in this study. Data sharing is not applicable to this article.

## Abbreviations

The following abbreviations are used in this manuscript:

AAV	adeno-associated virus
AAV6	adeno-associated virus serotype 6
Cas	CRISPR-associated protein
Cas9	CRISPR-associated protein 9
CRISPR	clustered regularly interspaced short palindromic repeats
CRISPRa	CRISPR activation
CRISPRi	CRISPR interference
crRNA	CRISPR RNA
dCas9	nuclease-dead Cas9
DISCOVER-seq	discovery of in situ Cas off-targets and verification by sequencing
DNA	deoxyribonucleic acid
DSB	double-strand break
EV	extracellular vesicle
GUIDE-seq	genome-wide unbiased identification of DSBs enabled by sequencing
HDR	homology-directed repair
HLA	human leukocyte antigen
KRAB	Krüppel-associated box
MHC	major histocompatibility complex
MISEV2023	Minimal Information for Studies of Extracellular Vesicles 2023
mRNA	messenger RNA
MSC	mesenchymal stem cell
MSC-EV	MSC-derived extracellular vesicle
NHEJ	non-homologous end joining
NK	natural killer
PAM	protospacer-adjacent motif
RNA	ribonucleic acid
RNP	ribonucleoprotein
SAM	synergistic activation mediator
sgRNA	single-guide RNA
SpCas9	Streptococcus pyogenes Cas9
TALEN	transcription activator-like effector nuclease
tracrRNA	trans-activating CRISPR RNA
VP64	four tandem copies of the VP16 transcriptional activation domain
VPR	VP64–p65–Rta tripartite transcriptional activator
ZFN	zinc-finger nuclease
